# Reviewer List

**DOI:** 10.1093/ijnp/pyae008

**Published:** 2024-02-16

Listed below are the reviewers for calendar year 2023. We sincerely appreciate their reviewing for the

*International Journal of Neuropsychopharmacology.*

*Most Reviews Submitted in a Timely Manner

Abdelraouf, Sahar M.

Albert, Paul

Amat, Jose

Anokhin, Andrey

Antonucci, Flavia

Arakawa, Ryosuke

Banghart, Matthew

Baratta, Michael

Berridge, Craig

Berridge, Kent

Brašic´, James

Brudzynski, Stefan

Campbell, Megan Loraine

Carreno, Flavia

Chadha, Radhika

Chan, Kenny

Cheng, Yong

Chidharom, Matthieu

Clarke, William

Codeluppi, Sierra

Cohen, Phillip

Collins, Greg

Cumming, Paul

Di Miceli, Mathieu

Ding, Junjie

Donegan, Jennifer

Dong, Yan

Dwivedi, Yogesh

Elam, Hannah

Forero, Diego

Fremont, Rachel

Fucich, Elizabeth

Furuyashiki, Tomoyuki

Galfalvy, Hanga

Gambarana, Carla

Georgiou, Polymnia

Gilman, Lee

Ginsburg, Brett

*Girgis, Ragy

*Girotti, Milena

Gonzalez-Maeso, Javier

Gould, Georgianna

Grimm, Jeff

Gronier, Benjamin

Grover, Sandeep

Grunebaum, Michael

Gururajan, Anand

Guterstam, Joar

Hantsoo, Liisa

*Hashimoto, Kenji

Hersey, Melinda

Ho, Roger C.

Hosseini, Shirin

Howland, John

Hwa, Lara

Issler, Orna

Ivanov, Iliyan

Jha, Manish

Johnston, Jenessa

Kamatham, Pushpa Tryphena

Kantrowitz, Joshua

Katona, Istvan

Keedy, Sarah

Kelly, Michy

Kim, Helena

Kimoto, Sohei

Kirschner, Matthias

Klein, Matthew

Knackstedt, Lori

Kohtala, Samuel

Koike, Shinsuke

Korade, Zelijka

Kwan, Alex

Li, Chiang-shan

Li, Jun-Xu

Li, Yanhui

Lindenmayer, Jean-Pierre

Liu, Chun-Hong

Lodge, Daniel

Lutfy, Kabirullah

Maguire, David

Maunder, Robert

McVoy, Molly

Melhem, Nadine

Mouchlianitis, Elias

Nagai, Hirotaka

Nakajima, Shinichiro

Newcorn, Jeff

Nichols, Charles

Nordahl, Thomas

Northoff, Georg

Orsini, Caitlin

Ortega, Miquel

Ortuno, Felipe

Paredes, Denisse

Parry, Barbara

Parsons, Ryan

Petridis, Petros

Pistis, Marco

Radley, Jason

Rae, Mariana

Raskind, Murray

Reppucci, Christina

Rieger, Nathan

Rivi, Veronica

Rodriguez, Rodrigo

Roy, Bhaskar

Ruzza, Chiara

Sahebi, Ali

Salech, Felipe

Sartorius, Alexander

Schwarting, Rainer

Sheffield, Julia

Shishmanova-Doseva, Michaela

Shukla, Rammohan

Sidana, Ajeet

Simola, Nicola

Sinclair, Lindsey

Smith, Andrew

Sumiyoshi, Tomiki

Susai, Subash Raj

Suzuki, Takefumi

Szymkowicz, Sarah

Tanquturi, Parthasaradhireddy

Tanzer, Tim

Tavakoli, Paniz

Thanos, Panayotis

Thomas, Kristen

Torrens, Alexa

Tseng, Kuei

Usiello, Alessandro

Vanmierlo, Tim

Varney, M.A.

Weiss, Jay

Whiskey, Eromona

Yang, Sheng-tao

Young, Jared

Zanos, Panos

Zhang, Yun-Feng

Zhilyaeva, T.

